# Supplementary material for: Novel phages of Pseudomonas syringae unveil numerous potential auxiliary metabolic genes
Source: J Gen Virol. 2024 Jun 4;105(6):001990. doi: 10.1099/jgv.0.001990 (PMC11256456; doi:10.1099/jgv.0.001990)
Supplement: Uncited Supplementary Material 1. [file jgv-105-01990-s001.pdf]

Novel phages of *Pseudomonas syringae* unveil numerous potential auxiliary metabolic genes

Chloé Feltin[1*], Julian R. Garneau[2], Cindy E. Morris[1], Annette Bérard[3], Clara Torres-Barceló[1*]

[*] Corresponding authors

Supp Table 1 to 3

Supp Figure 1 to 5

Supp Table 1: Characteristics of the orchards used to sample the phage collection.

Orchard name	Variety	Grafting	Importance of symptoms in the orchard	Treatments used
Hybrid 2015/2016	Hybrid	No grafting	High	Against ACL*
CaPReD	Anégat / Frisson	Peach tree (Monclar) at 120 cm	Medium	Without treatment
Core Collection	150 varieties	Apricot tree (Manicot) at 60 cm	Depends on variety	Without treatment

*ACL = Apricot chlorotic leafroll caused by a phytoplasm treated by insecticides.

Supp Table 2: Phages referenced in GenBank that are the most closely related to the phages of *P. syringae* in our collection.

Genera	Species	Clones	Clostest hits	Percent identity	Query Coverage	GenBank reference
Aravirus	*cervantes*	Arace01	Pseudomonas phage Zuri	84.33%	0.14	NC_049456.2
Aurigavirus	*capella*	Aurca01	Pseudomonas phage Medea1	96.60%	0.35	MW862109.1
Cruxvirus	*imai*	Cruim01	*Pseudomonas sy*	92.82%	0.27	CP047072.1
Cygnusvirus	*sadr*	Cygsa01	Pseudomonas phage nickie	72.82%	0	NC_042091.1
Dracovirus	*altais*	Draal01	Pseudomonas phage Misse	76.57%	0.34	MW286267.1
Dracovirus	*altais*	Draal02	Pseudomonas phage Bertil	80.21%	0.37	MW286266.1
Dracovirus	*altais*	Draal03	Pseudomonas phage Misse	76.57%	0.3	MW286267.1
Dracovirus	*eltanin*	Drael01	Pseudomonas phage Misse	76.57%	0.35	MW286267.1
Ghunavirus	*alcor*	Ghual01	Pseudomonas phage MR1	93.46%	0.91	MT104465.1
Ghunavirus	*chalawan*	Ghuch01	Pseudomonas phage MR1	94.57%	0.9	MT104465.1
Lepusvirus	*nihal*	Lepni01	Pseudomonas phage Henninger	79.29%	0.47	NC_047922.1
Lyravirus	*sulafat*	Lyrsu01	Pseudomonas phage UFV-P2	73.41%	0.01	NC_018850.2
Lyravirus	*sulafat*	Lyrsu02	Pseudomonas phage UFV-P2	73.41%	0.01	NC_018850.2
Lyravirus	*sulafat*	Lyrsu03	Pseudomonas phage UFV-P2	73.41%	0.01	NC_018850.2
Nickievirus	*ankaa*	Nican01	Pseudomonas phage phiK7B1	87.68%	0.89	MT354569.1
Orionvirus	*mintaka*	Orimi01	Pseudomonas phage MR15	92.64%	0.02	MT104475.1
Orionvirus	*saiph*	Orisa01	Pseudomonas phage MR15	92.64%	0.02	MT104475.1
Orionvirus	*saiph*	Orisa02	Pseudomonas phage MR15	92.64%	0.02	MT104475.1
Orionvirus	*saiph*	Orisa03	Pseudomonas phage MR14	95.83%	0	MT104474.1
Pavovirus	*peacock*	Pavpe01	Escherichia phage Halfdan	75.87%	0.78	MH362766.1
Pyxisvirus	*pyxidis*	Pyxpy01	Pseudomonas phage PN09	80.31%	0.42	MW175491.1
Pyxisvirus	*pyxidis*	Pyxpy02	Pseudomonas phage PN09	80.31%	0.42	MW175491.1
Toucanavirus	*emiw*	Touem01	Pseudomonas phage Dolphis	78.48%	0.08	MT711888.1
Uliginvirus	*naos*	Ulina01	Pseudomonas phage BUCT553	94.31%	0.97	MT941682.1
Uliginvirus	*tureis*	Ulitu01	Pseudomonas phage BUCT553	94.82%	0.97	MT941682.1

For each phage in the collection, their proximity to a reference phage is estimated by the percent identity and the percent of query coverage. GenBank references are those of phages close to the collection.

Supp Table 3: Predicted lifestyle of *P. syringae* phages and identified gene function related to the lysogenic cycle.

Genera	Species	Clones	Prediction accuracy (%)	Predicted lifestyle	Genes related to lysogenic cycle
Aravirus	*cervantes*	Arace01	87.86	Virulent	-
Aurigavirus	***capella***	**Aurca01**	**76.09**	**Temperate**	**Integrase, repressor protein CI, Cro-like protein, putative transposase**
Cruxvirus	***imai***	**Cruim01**	**71.77**	**Temperate**	**Integrase, repressor protein CII**
Cygnusvirus	*sadr*	Cygsa01	96.44	Virulent	-
Dracovirus	*altais*	Draal01	99.71	Virulent	-
Dracovirus	*altais*	Draal02	100	Virulent	-
Dracovirus	*altais*	Draal03	100	Virulent	-
Dracovirus	*eltanin*	Drael01	99.7	Virulent	-
Ghunavirus	*alcor*	Ghual01	97.75	Virulent	-
Ghunavirus	*chalawan*	Ghuch01	98.98	Virulent	-
Lepusvirus	*nihal*	Lepni01	97.83	Virulent	-
Lyravirus	*sulafat*	Lyrsu01	100	Virulent	-
Lyravirus	*sulafat*	Lyrsu02	100	Virulent	-
Lyravirus	*sulafat*	Lyrsu03	100	Virulent	-
Nickievirus	*ankaa*	Nican01	83.26	Virulent	-
Orionvirus	*mintaka*	Orimi01	80.02	Virulent	-
Orionvirus	*saiph*	Orisa01	80.79	Virulent	-
Orionvirus	*saiph*	Orisa02	83.42	Virulent	-
Orionvirus	*saiph*	Orisa03	80.99	Virulent	-
Pavovirus	*peacock*	Pavpe01	99.1	Virulent	-
Pyxisvirus	*pyxidis*	Pyxpy01	96.03	Virulent	-
Pyxisvirus	*pyxidis*	Pyxpy02	95.04	Virulent	-
Toucanavirus	***emiw***	**Touem01**	**89.32**	**Temperate**	**Integrase, ParA-like protein**
Uliginvirus	*naos*	Ulina01	99.5	Virulent	-
Uliginvirus	*tureis*	Ulitu01	99.55	Virulent	-

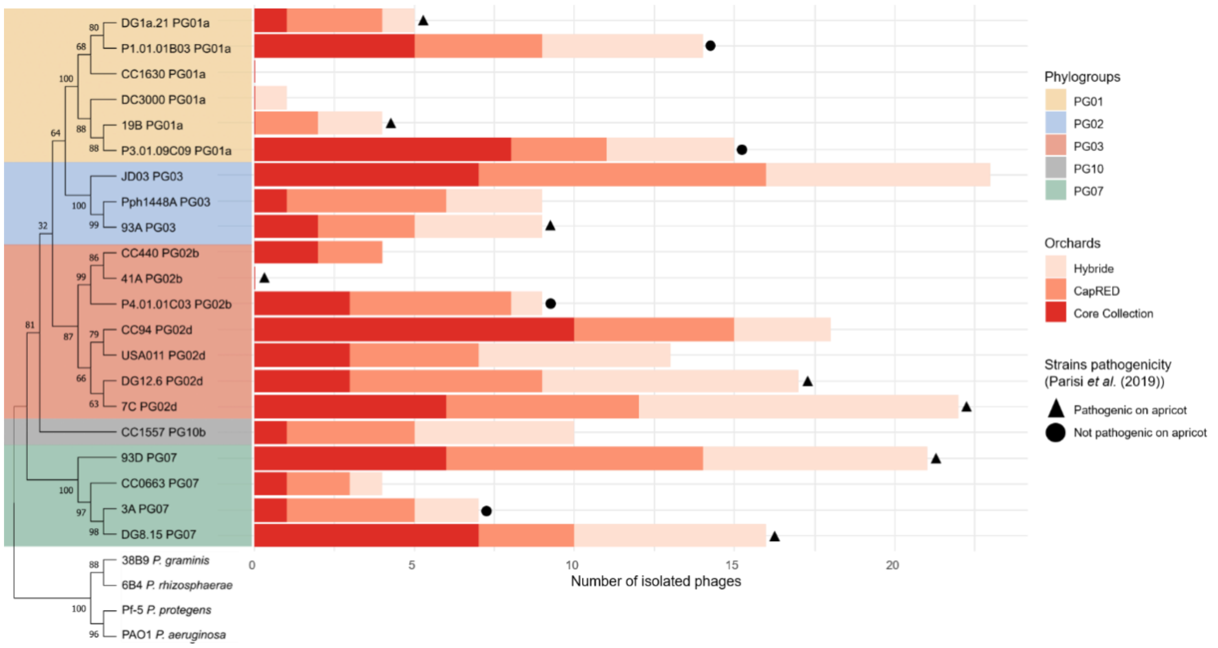

Supp Figure 1: Origin of the 221 phage isolates according to the *P. syringae* strains and the three orchards. The P. syringae strains are represented in a Neighbor Joining tree from the housekeeping cts (citrate synthase) gene and rooted with strains outside of the species complex. The four phylogroups in this tree are delimited by different coloured backgrounds (yellow: PG01a, blue: PG03, pink: PG02b and d, grey: PG10b, green: PG07). The colours of the bars represent the three orchards. The black triangles show the strains pathogenic on apricot; and the black circles indicate the strains that were not pathogenic on apricot (Parisi *et al.* 2019). Strains not marked with a triangle or circle were not tested for pathogenicity on apricot.

Genera	Species	Clones	Orimi01	Orisa03	Orisa02	Orisa01	Cruim01	Aurca01	Cygsa01	Pseudomonas phage nickie	Nican01	Pseudomonas phage phiK7B1	Lepni01	Pseudomonas phage Henninger	Ghual01	Pseudomonas phage MR1	Ghuch01	Lyrsu03	Lyrsu01	Lyrsu02	Arace01	Ulina01	Ulitu01	Pseudomonas phage BUCT553	Drael01	Draal01	Draal03	Draal02	Pyxpy01	Pyxpy02	Pavpe01	Touem01
Orionvirus	*mintaka*	Orimi01	100,0																													
	saiph	Orisa03	91,0	100,0																												
		Orisa02	94,7	95,5	100,0																											
		Orisa01	95,0	95,9	99,6	100,0																										
Cruxvirus	*imai*	Cruim01	3,3	2,4	2,5	2,5	100,0																									
Aurigavirus	*capella*	Aurca01	3,0	2,6	3,0	3,0	68,9	100,0																								
Cygnusvirus	*sadr*	Cygsa01	0,1	0,1	0,1	0,1	0,0	0,1	100,0																							
Nickievirus	-	Pseudomonas phage nickie	0,0	0,0	0,0	0,0	0,0	0,1	1,4	100,0																						
	ankaa	Nican01	0,0	0,0	0,0	0,0	0,0	0,1	1,2	70,0	100,0																					
	-	Pseudomonas phage phiK7B1	0,0	0,0	0,0	0,0	0,0	0,1	1,2	70,2	80,6	100,0																				
Lepusvirus	*nihal*	Lepni01	0,0	0,0	0,0	0,0	0,0	0,0	0,0	0,0	0,0	0,0	100,0																			
Ghunavirus	-	Pseudomonas phage Henninger	0,1	0,1	0,1	0,1	0,0	0,0	0,1	0,0	0,0	0,0	58,4	100,0																		
	alcor	Ghual01	0,0	0,0	0,0	0,0	0,0	0,0	0,0	0,0	0,1	0,0	58,8	71,7	100,0																	
	-	Pseudomonas phage MR1	0,0	0,0	0,0	0,0	0,0	0,0	0,0	0,0	0,0	0,0	57,7	73,6	87,7	100,0																
	chalawan	Ghuch01	0,0	0,0	0,0	0,0	0,0	0,0	0,0	0,0	0,0	0,0	58,7	75,1	88,3	89,1	100,0															
Lyravirus	*sulafat*	Lyrsu03	0,0	0,0	0,0	0,0	0,0	0,0	0,1	0,5	0,6	0,7	0,1	0,1	0,3	0,1	0,2	100,0														
		Lyrsu01	0,0	0,0	0,0	0,0	0,0	0,0	0,1	0,5	0,6	0,7	0,1	0,1	0,3	0,1	0,2	99,2	100,0													
		Lyrsu02	0,0	0,0	0,0	0,0	0,0	0,0	0,1	0,5	0,6	0,7	0,1	0,1	0,3	0,1	0,2	99,1	99,9	100,0												
Aravirus	*cervantes*	Arace01	0,0	0,0	0,0	0,0	0,2	0,2	0,0	0,0	0,2	0,1	0,0	0,0	0,0	0,0	0,0	0,0	0,0	0,0	100,0											
Uliginvirus	*naos*	Ulina01	0,0	0,0	0,0	0,0	0,0	0,0	0,0	0,0	0,0	0,1	0,4	0,3	0,6	0,6	0,6	0,2	0,2	0,2	1,1	100,0										
	tureis	Ulitu01	0,0	0,0	0,0	0,0	0,0	0,0	0,0	0,0	0,0	0,0	0,5	0,2	0,7	0,8	0,6	0,3	0,3	0,3	0,8	93,4	100,0									
	-	Pseudomonas phage BUCT553	0,0	0,0	0,0	0,0	0,0	0,0	0,0	0,0	0,0	0,1	0,6	0,7	0,5	0,3	0,4	0,3	0,3	0,3	0,8	92,5	93,9	100,0								
Dracovirus	*eltanin*	Drael01	0,0	0,0	0,0	0,0	0,0	0,0	0,0	0,0	0,0	0,0	0,1	0,0	0,1	0,0	0,0	0,3	0,3	0,3	0,1	1,9	1,8	1,8	100,0							
	altais	Draal01	0,0	0,0	0,0	0,0	0,0	0,0	0,0	0,0	0,0	0,0	0,1	0,0	0,1	0,0	0,0	0,0	0,0	0,0	0,1	1,8	1,8	1,8	94,8	100,0						
		Draal03	0,0	0,0	0,0	0,0	0,0	0,0	0,0	0,0	0,0	0,0	0,1	0,0	0,1	0,0	0,0	0,0	0,0	0,0	0,1	1,8	1,8	1,8	94,8	99,9	100,0					
		Draal02	0,0	0,0	0,0	0,0	0,0	0,0	0,0	0,0	0,0	0,0	0,1	0,0	0,1	0,0	0,0	0,0	0,0	0,0	0,1	1,8	1,8	1,8	94,8	99,9	99,9	100,0				
Pyxisvirus	*pyxidis*	Pyxpy01	0,0	0,0	0,0	0,0	0,0	0,0	0,0	0,0	0,0	0,0	0,0	0,1	0,1	0,1	0,1	0,2	0,2	0,2	0,2	0,0	0,0	0,0	0,2	0,2	0,2	0,2	100,0			
		Pyxpy02	0,0	0,0	0,0	0,0	0,0	0,0	0,0	0,0	0,0	0,0	0,0	0,1	0,1	0,1	0,1	0,2	0,2	0,2	0,2	0,0	0,0	0,0	0,2	0,2	0,2	0,2	99,1	100,0		
Pavovirus	*peacock*	Pavpe01	0,0	0,0	0,0	0,0	0,0	0,0	0,0	0,0	0,0	0,0	0,0	0,1	0,0	0,0	0,0	0,0	0,0	0,0	0,5	0,2	0,2	0,2	0,0	0,0	0,0	0,0	0,0	0,0	100,0	
Toucanavirus	*emiw*	Touem01	0,7	0,9	0,7	0,7	1,8	1,8	0,1	0,0	0,1	0,0	0,0	0,0	0,0	0,0	0,0	0,0	0,0	0,0	0,0	0,0	0,0	0,0	0,0	0,0	0,0	0,0	0,0	0,0	0,0	100,0

Supp Figure 2: Genetic distance matrix of the 25 *P. syringae* phages compared to the 5-close reference phages (in yellow), produced with VIRIDIC. The heatmap displays the percentage of inter-genome identity and phage genera clusters are indicated in green and in the first columns (species and clones are included for clarity purposes).

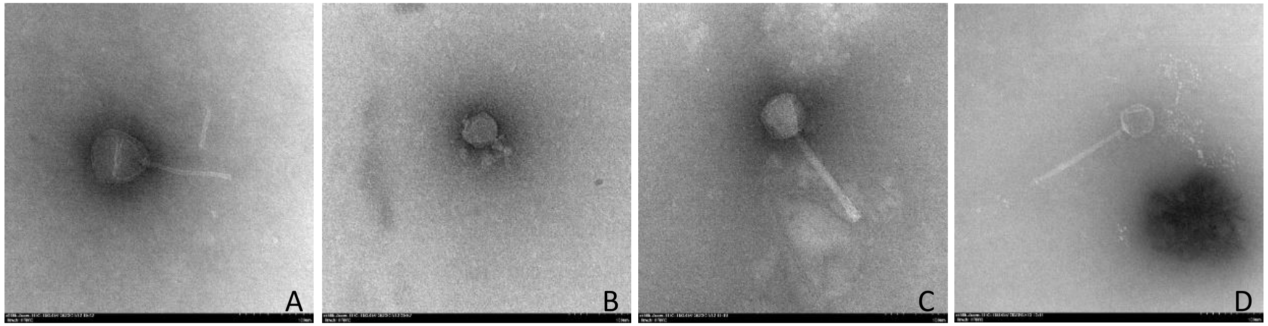

Supp Figure 3: TEM image of 4 *P. syringae* phages presenting three different morphologies: siphovirus (A, virulent phage Nican01), podovirus (B, virulent phage Lyrsu02), myovirus (C, temperate phage Touem01) and siphovirus (D, virulent phage Pavpe01).

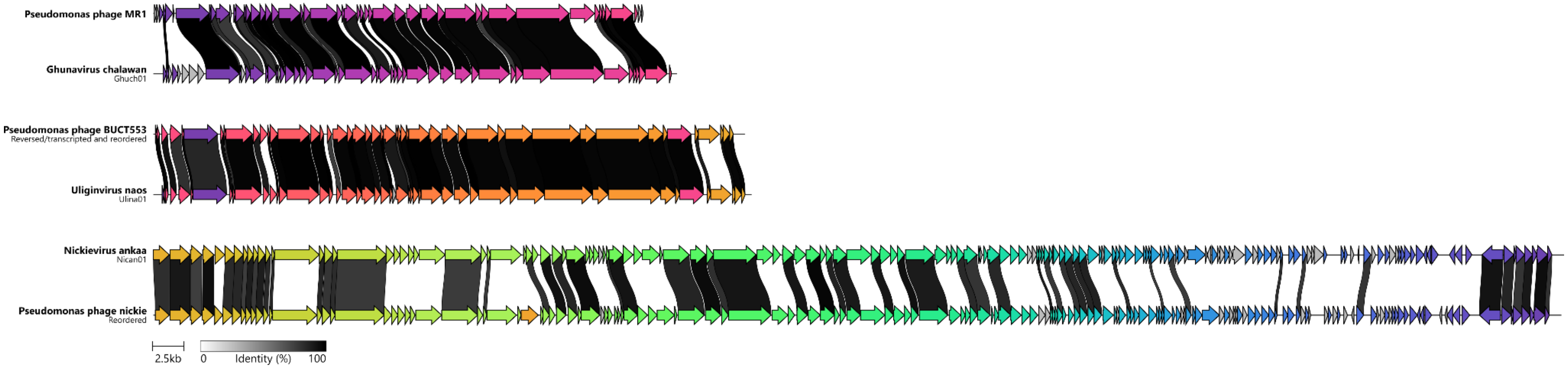

Supp Figure 4: Genomic comparison of the 3 phage genera previously described with the 3 new *P. syringae* phages belonging to them using Clinker. The genomes previously described have been reorganised or reversed to allow alignment with the new genomes. The arrows indicate the open reading frames and their direction. Link identity are presented as black to white bands and threshold was set to 80% protein identity.

Supp Figure 5: Reports on the PhageTerm analysis of the three clones of the *Lyravirus sulafat* species.

Lyravirus sulafat Lyrsu01 PhageTerm Analysis

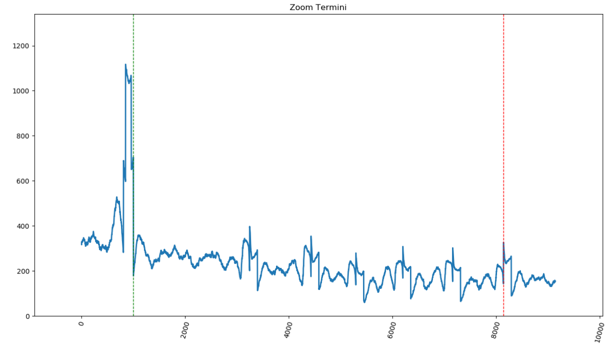

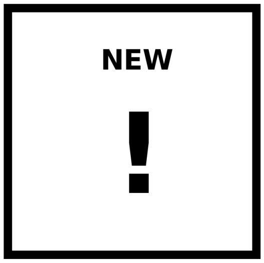

PhageTerm Method

Ends	Left (red)	Right (green)	Permuted	Orientation	Class	Type
Redundant	20466	13323	No	NA	-	-

Strand	Location	T	pvalue
+	20466	0.93	3.34e-51
	26881	0.92	2.82e-43
	39573	0.91	1.87e-51
	18525	0.90	7.09e-40
	17618	0.89	9.66e-39
-	13323	0.79	4.94e-181
	11282	0.26	5.55e-15
	6133	0.24	3.64e-04
	4405	0.14	2.12e-03
	972	0.12	9.51e-03

T (Start. Pos. Cov. / Whole Cov.)

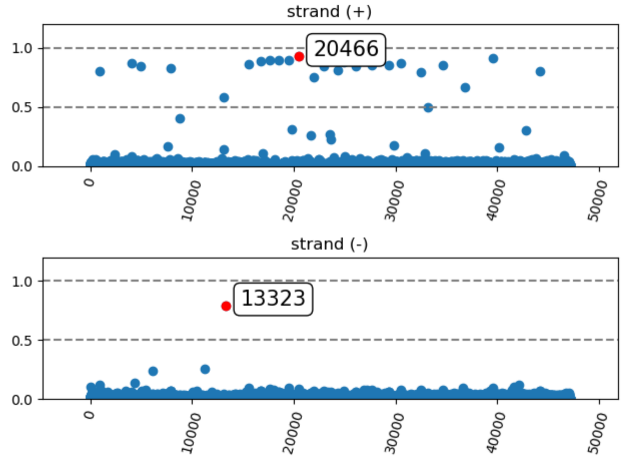

Li's Method

Packaging	Termini	Forward	Reverse	Orientation
PAC	Fixed	Multiple-Pref. Term.	Obvious Termini	Reverse

Strand	Location	SPC	R
+	13133	369	2.0
	7900	226	-
	4996	204	-
	956	196	-
	15568	193	-
-	13323	525	11.0
	11282	50	-
	6133	28	-
	4405	19	-
	972	17	-

SPC

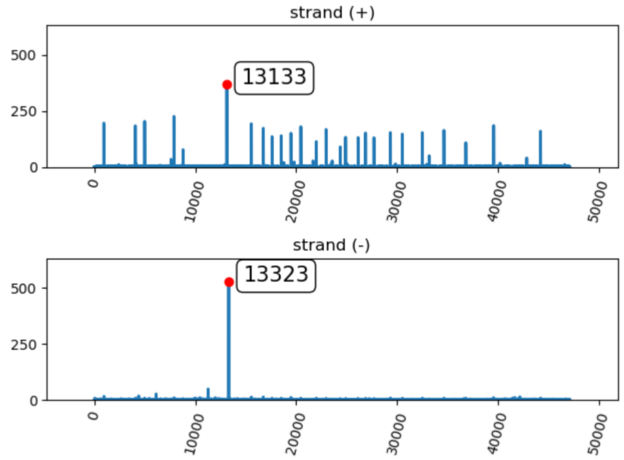

Analysis Methodology

PhageTerm software uses raw reads of a phage sequenced with a sequencing technology using random fragmentation and its genomic reference sequence to determine the termini position. The process starts with the alignment of NGS reads to the phage genome in order to calculate the starting position coverage (SPC), where a hit is given only to the position of the first base in a successfully aligned read (the alignment algorithm uses the lenght of the seed (default: 20) for mapping and does not accept gap or missmatch to speed up the process). Then the program apply 2 distinct scoring methods: i) a statistical approach based on the Gamma law; and ii) a method derived from LI and al. 2014 paper.

General set-up and mapping informations

**Phage Genome**	47123 bp
**Sequencing Reads**	70937
**Mapping Reads**	97 %
OPTIONS	
Mapping Seed	*20*
Surrounding	*20*
Host Analysis	*No*

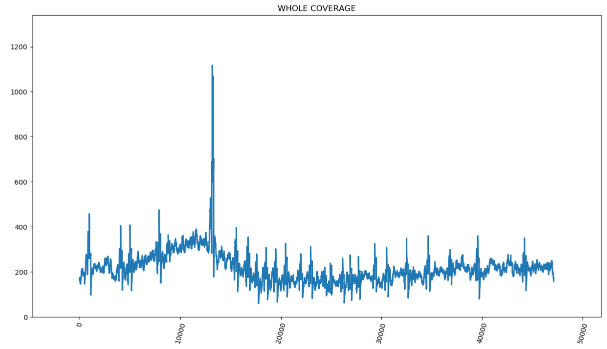

Highest peak of each side coverage graphics

**Whole Coverage Zoom (Left)**	**Whole Coverage Zoom (Right)**

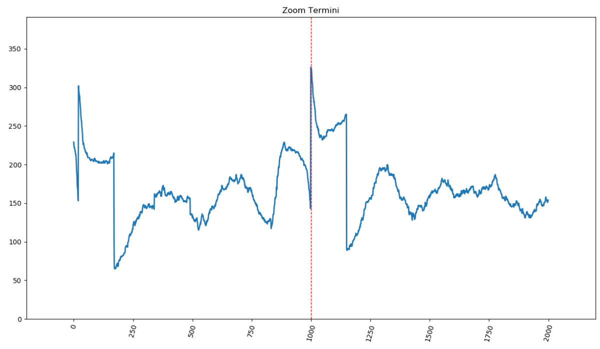

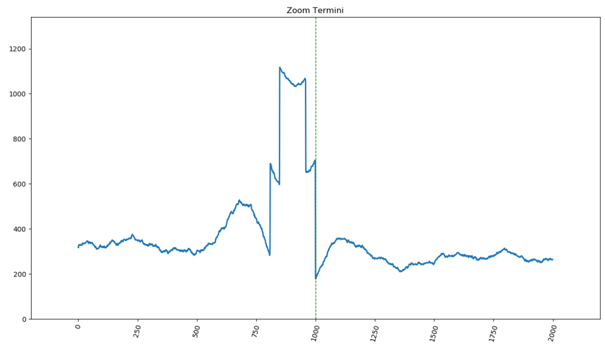

General controls information

**Whole genome coverage**	108	WARNING: Low (<200), Li's method could not be reliable
**Weak genome coverage**	0.0 %	OK
**Reads lost during alignment**	2.4 %	OK

i) PhageTerm method

Reads are mapped on the reference to determine the starting position coverage (SPC) as well as the coverage (COV) in each orientation. These values are then used to compute the variable T = SPC / COV. The average value of T at positions along the genome that are not termini is expected to be 1/F, where F is the average fragment size. For the termini that depends of the packaging mode. Cos Phages: no reads should start before the terminus and therefore X=1. DTR phages: for N phages present in the sample, there should be N fragments that start at the terminus and N fragments that cover the edge of the repeat on the other side of the genome as a results T is expected to be 0.5. Pac phages: for N phages in the sample, there should be N/C fragments starting at the pac site, where C is the number of phage genome copies per concatemer. In the same sample N fragments should cover the pac site position, T is expected to be (N/C)/(N+N/C) = 1/(1+C). To assess whether the number of reads starting at a given position along the genome can be considered a significant outlier, PhageTerm first segments the genome according to coverage using a regression tree. A gamma distribution is then fitted to SPC for each segment and an adjusted p-value is computed for each position. Finally if several significant peaks are detected within a small sequence window (default: 20bp), their T values are merged.

**Nearby Termini (Forward / Reverse)**	0 / 0	Peaks localized 20 bases around the maximum

ii) Li's method

The second approach is based on the calculation and interpretation of three specific ratios R1, R2 and R3 as suggested in a previous publication from Li et al. 2014. The first ratio, is calculated as follow: the highest starting frequency found on either the forward or reverse strands is divided by the average starting frequency, R1 = (highest frequency/average frequency). Li's et al. have proposed three possible interpretation of the R1 ratio. First, if R1 < 30, the phage genome does not have any termini, and is either circular or completely permuted and terminally redundant. The second interpretation for R1 is when 30 ≤ R1 ≥ 100, suggesting the presence of preferred termini with terminal redundancy and apparition of partially circular permutations. At last if R1 > 100 that is an indication that at least one fixed termini is present with terminase recognizing a specific site. The two other ratios are R2 and R3 and the calculation is done in a similar manner. R2 is calculated using the highest two frequencies (T1-F and T2-F) found on the forward strand and R3 is calculated using the highest two frequencies (T1-R and T2-R) found on the reverse strand. To calculate these two ratios, we divide the highest frequency by the second highest frequency T2. So R2 = (T1-F / T2-F) and R3 = (T1-R / T2-R). These two ratios are used to analyze termini characteristics on each strand taken individually. Li et al. suggested two possible interpretations for R2 and R3 ratios combine to R1. When R1 < 30 and R2 < 3, we either have no obvious termini on the forward strand, or we have multiple preferred termini on the forward strand, if 30 ≤ R1 ≤ 100. If R2 > 3, it is suggested that there is an obvious unique termini on the forward strand. The same reasoning is applicable for the result of R3. Combining the results for ratios found with this approach, it is possible to make the first prediction for the viral packaging mode of the analyzed phage. A unique obvious termini present at both ends (both R2 and R3 > 3) reveals the presence of a COS mode of packaging. The headful mode of packaging PAC is concluded when we have a single obvious termini only on one strand. A whole coverage around 500X is needed for this method to be reliable.

**Nearby Termini (Forward / Reverse)**	0 / 0	Peaks localized 20 bases around the maximum
**R1 - highest freq./average freq.**	714	At least one fixed termini is present with terminase recognizing a specific site.
**R2 Forw - highest freq./second freq.**	2	Multiple preferred termini on the forward strand.
**R3 Rev - highest freq./second freq.**	11	Unique termini on the reverse strand.

Lyravirus sulafat Lyrsu02 PhageTerm Analysis

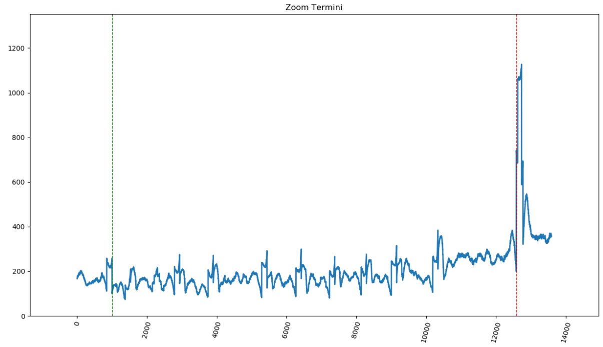

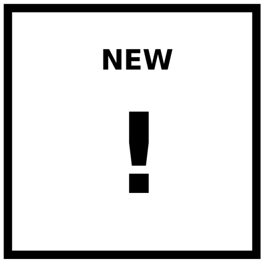

PhageTerm Method

Ends	Left (red)	Right (green)	Permuted	Orientation	Class	Type
Redundant	37641	26058	No	NA	-	-

Strand	Location	T	pvalue
+	37641	0.79	2.56e-131
	39682	0.29	1.72e-12
	44831	0.23	4.16e-07
	46559	0.16	1.82e-02
	8800	0.15	8.07e-08
-	26058	0.92	4.22e-44
	30498	0.91	4.59e-48
	34211	0.89	2.62e-62
	16324	0.88	2.71e-48
	32439	0.88	5.01e-35

T (Start. Pos. Cov. / Whole Cov.)

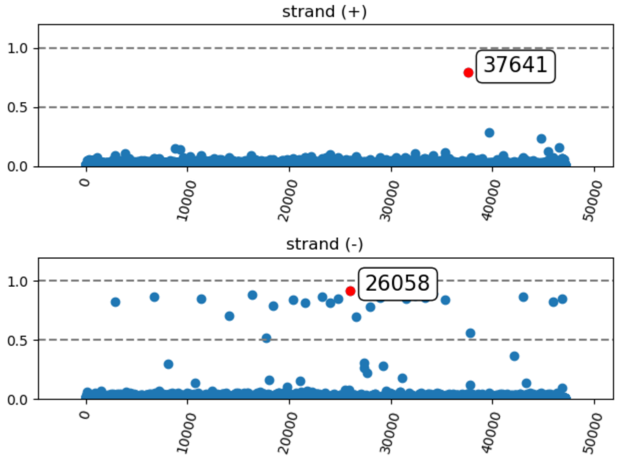

Li's Method

Packaging	Termini	Forward	Reverse	Orientation
PAC	Fixed	Obvious Termini	Multiple-Pref. Term.	Forward

Strand	Location	SPC	R
+	37641	541	9.0
	39682	60	-
	44831	36	-
	46559	23	-
	8800	21	-
-	37831	339	1.0
	43064	239	-
	45968	202	-
	6751	172	-
	2940	171	-

SPC

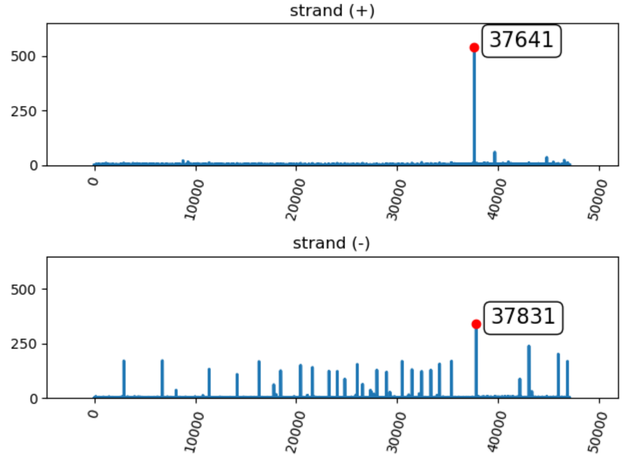

Analysis Methodology

PhageTerm software uses raw reads of a phage sequenced with a sequencing technology using random fragmentation and its genomic reference sequence to determine the termini position. The process starts with the alignment of NGS reads to the phage genome in order to calculate the starting position coverage (SPC), where a hit is given only to the position of the first base in a successfully aligned read (the alignment algorithm uses the lenght of the seed (default: 20) for mapping and does not accept gap or missmatch to speed up the process). Then the program apply 2 distinct scoring methods: i) a statistical approach based on the Gamma law; and ii) a method derived from LI and al. 2014 paper.

General set-up and mapping informations

**Phage Genome**	47123 bp
**Sequencing Reads**	71442
**Mapping Reads**	97 %
OPTIONS	
Mapping Seed	*20*
Surrounding	*20*
Host Analysis	*No*

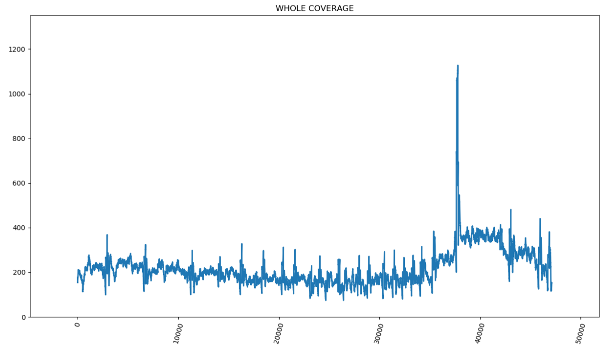

Highest peak of each side coverage graphics

**Whole Coverage Zoom (Left)**	**Whole Coverage Zoom (Right)**

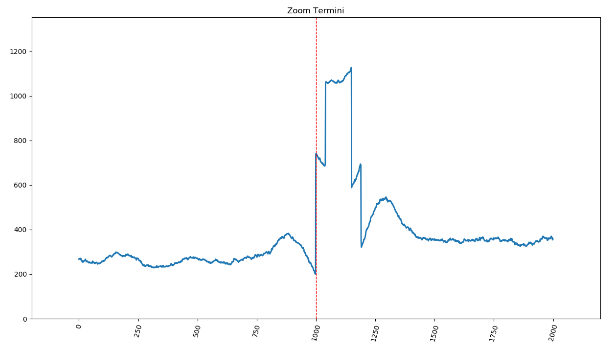

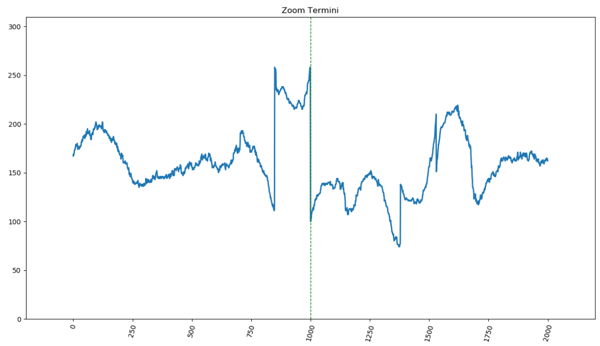

General controls information

**Whole genome coverage**	108	WARNING: Low (<200), Li's method could not be reliable
**Weak genome coverage**	0.0 %	OK
**Reads lost during alignment**	2.7 %	OK

i) PhageTerm method

Reads are mapped on the reference to determine the starting position coverage (SPC) as well as the coverage (COV) in each orientation. These values are then used to compute the variable T = SPC / COV. The average value of T at positions along the genome that are not termini is expected to be 1/F, where F is the average fragment size. For the termini that depends of the packaging mode. Cos Phages: no reads should start before the terminus and therefore X=1. DTR phages: for N phages present in the sample, there should be N fragments that start at the terminus and N fragments that cover the edge of the repeat on the other side of the genome as a results T is expected to be 0.5. Pac phages: for N phages in the sample, there should be N/C fragments starting at the pac site, where C is the number of phage genome copies per concatemer. In the same sample N fragments should cover the pac site position, T is expected to be (N/C)/(N+N/C) = 1/(1+C). To assess whether the number of reads starting at a given position along the genome can be considered a significant outlier, PhageTerm first segments the genome according to coverage using a regression tree. A gamma distribution is then fitted to SPC for each segment and an adjusted p-value is computed for each position. Finally if several significant peaks are detected within a small sequence window (default: 20bp), their T values are merged.

**Nearby Termini (Forward / Reverse)**	0 / 0	Peaks localized 20 bases around the maximum

ii) Li's method

The second approach is based on the calculation and interpretation of three specific ratios R1, R2 and R3 as suggested in a previous publication from Li et al. 2014. The first ratio, is calculated as follow: the highest starting frequency found on either the forward or reverse strands is divided by the average starting frequency, R1 = (highest frequency/average frequency). Li's et al. have proposed three possible interpretation of the R1 ratio. First, if R1 < 30, the phage genome does not have any termini, and is either circular or completely permuted and terminally redundant. The second interpretation for R1 is when 30 ≤ R1 ≥ 100, suggesting the presence of preferred termini with terminal redundancy and apparition of partially circular permutations. At last if R1 > 100 that is an indication that at least one fixed termini is present with terminase recognizing a specific site. The two other ratios are R2 and R3 and the calculation is done in a similar manner. R2 is calculated using the highest two frequencies (T1-F and T2-F) found on the forward strand and R3 is calculated using the highest two frequencies (T1-R and T2-R) found on the reverse strand. To calculate these two ratios, we divide the highest frequency by the second highest frequency T2. So R2 = (T1-F / T2-F) and R3 = (T1-R / T2-R). These two ratios are used to analyze termini characteristics on each strand taken individually. Li et al. suggested two possible interpretations for R2 and R3 ratios combine to R1. When R1 < 30 and R2 < 3, we either have no obvious termini on the forward strand, or we have multiple preferred termini on the forward strand, if 30 ≤ R1 ≤ 100. If R2 > 3, it is suggested that there is an obvious unique termini on the forward strand. The same reasoning is applicable for the result of R3. Combining the results for ratios found with this approach, it is possible to make the first prediction for the viral packaging mode of the analyzed phage. A unique obvious termini present at both ends (both R2 and R3 > 3) reveals the presence of a COS mode of packaging. The headful mode of packaging PAC is concluded when we have a single obvious termini only on one strand. A whole coverage around 500X is needed for this method to be reliable.

**Nearby Termini (Forward / Reverse)**	0 / 0	Peaks localized 20 bases around the maximum
**R1 - highest freq./average freq.**	733	At least one fixed termini is present with terminase recognizing a specific site.
**R2 Forw - highest freq./second freq.**	9	Unique termini on the forward strand.
**R3 Rev - highest freq./second freq.**	1	Multiple preferred termini on the reverse strand.

Lyravirus sulafat Lyrsu03 PhageTerm Analysis

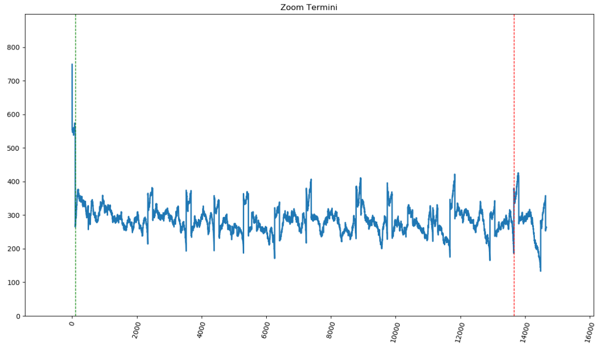

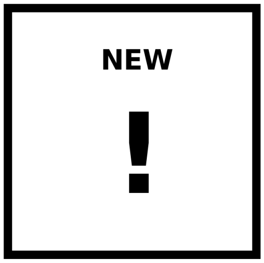

PhageTerm Method

Ends	Left (red)	Right (green)	Permuted	Orientation	Class	Type
Redundant	13655	97	No	NA	-	-

Strand	Location	T	pvalue
+	13655	0.83	3.59e-49
	38006	0.81	2.53e-48
	14484	0.81	4.00e-38
	6261	0.81	1.08e-38
	7240	0.80	2.16e-40
-	97	0.62	1.01e-119
	40058	0.15	4.12e-01
	45207	0.14	1.48e-02
	33241	0.13	1.00e+00
	33778	0.11	1.00e+00

T (Start. Pos. Cov. / Whole Cov.)

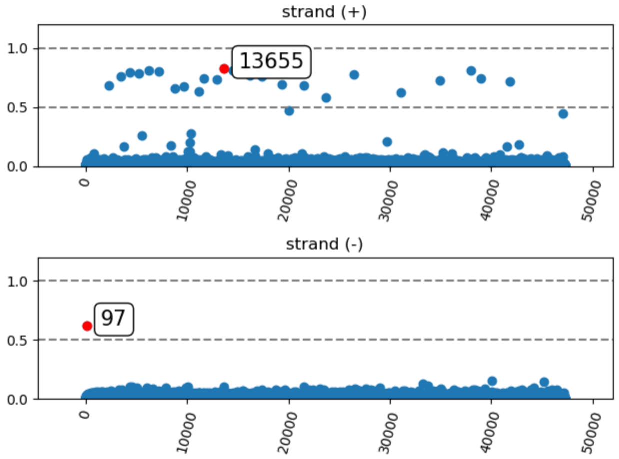

Li's Method

Packaging	Termini	Forward	Reverse	Orientation
PAC	Fixed	Multiple-Pref. Term.	Obvious Termini	Reverse

Strand	Location	SPC	R
+	47058	191	1.0
	16200	175	-
	13655	173	-
	17374	172	-
	38006	170	-
-	97	286	11.0
	45207	26	-
	40058	21	-
	4410	18	-
	33778	17	-

SPC

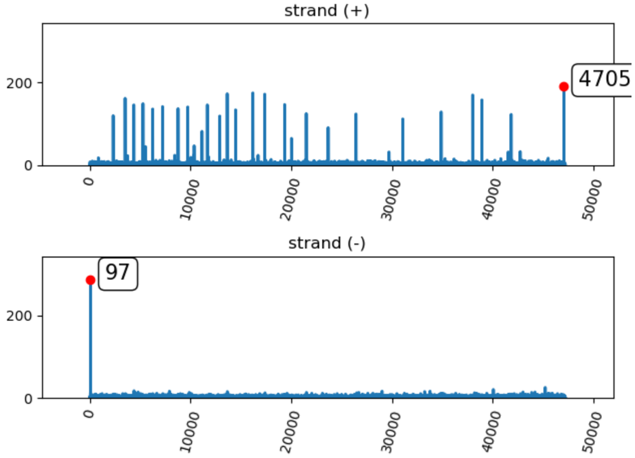

Analysis Methodology

PhageTerm software uses raw reads of a phage sequenced with a sequencing technology using random fragmentation and its genomic reference sequence to determine the termini position. The process starts with the alignment of NGS reads to the phage genome in order to calculate the starting position coverage (SPC), where a hit is given only to the position of the first base in a successfully aligned read (the alignment algorithm uses the lenght of the seed (default: 20) for mapping and does not accept gap or missmatch to speed up the process). Then the program apply 2 distinct scoring methods: i) a statistical approach based on the Gamma law; and ii) a method derived from LI and al. 2014 paper.

General set-up and mapping informations

**Phage Genome**	47206 bp
**Sequencing Reads**	97566
**Mapping Reads**	98 %
OPTIONS	
Mapping Seed	*20*
Surrounding	*20*
Host Analysis	*No*

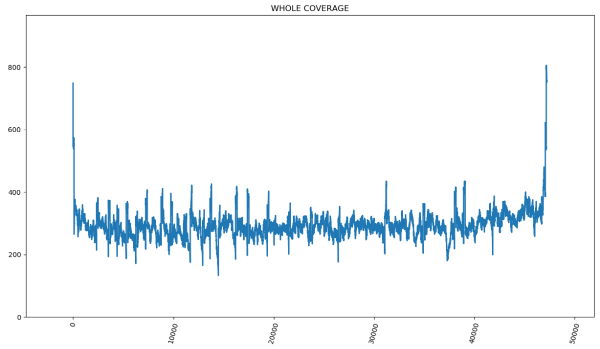

Highest peak of each side coverage graphics

Whole Coverage Zoom (Left) **Whole Coverage Zoom (Right)**

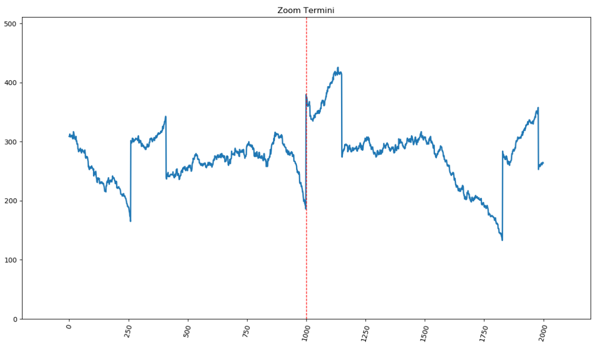

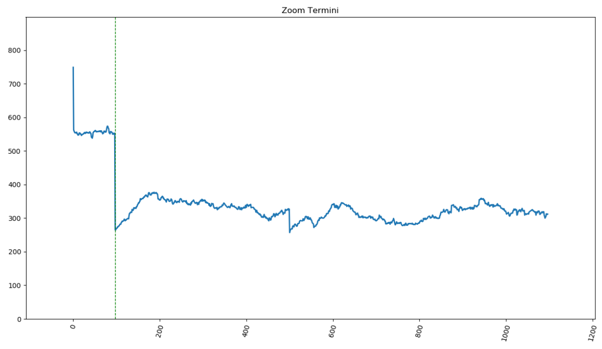

General controls information

**Whole genome coverage**	146	WARNING: Low (<200), Li's method could not be reliable
**Weak genome coverage**	0.0 %	OK
**Reads lost during alignment**	1.8 %	OK

i) PhageTerm method

Reads are mapped on the reference to determine the starting position coverage (SPC) as well as the coverage (COV) in each orientation. These values are then used to compute the variable T = SPC / COV. The average value of T at positions along the genome that are not termini is expected to be 1/F, where F is the average fragment size. For the termini that depends of the packaging mode. Cos Phages: no reads should start before the terminus and therefore X=1. DTR phages: for N phages present in the sample, there should be N fragments that start at the terminus and N fragments that cover the edge of the repeat on the other side of the genome as a results T is expected to be 0.5. Pac phages: for N phages in the sample, there should be N/C fragments starting at the pac site, where C is the number of phage genome copies per concatemer. In the same sample N fragments should cover the pac site position, T is expected to be (N/C)/(N+N/C) = 1/(1+C). To assess whether the number of reads starting at a given position along the genome can be considered a significant outlier, PhageTerm first segments the genome according to coverage using a regression tree. A gamma distribution is then fitted to SPC for each segment and an adjusted p-value is computed for each position. Finally if several significant peaks are detected within a small sequence window (default: 20bp), their T values are merged.

**Nearby Termini (Forward / Reverse)**	0 / 0	Peaks localized 20 bases around the maximum

ii) Li's method

The second approach is based on the calculation and interpretation of three specific ratios R1, R2 and R3 as suggested in a previous publication from Li et al. 2014. The first ratio, is calculated as follow: the highest starting frequency found on either the forward or reverse strands is divided by the average starting frequency, R1 = (highest frequency/average frequency). Li's et al. have proposed three possible interpretation of the R1 ratio. First, if R1 < 30, the phage genome does not have any termini, and is either circular or completely permuted and terminally redundant. The second interpretation for R1 is when 30 ≤ R1 ≥ 100, suggesting the presence of preferred termini with terminal redundancy and apparition of partially circular permutations. At last if R1 > 100 that is an indication that at least one fixed termini is present with terminase recognizing a specific site. The two other ratios are R2 and R3 and the calculation is done in a similar manner. R2 is calculated using the highest two frequencies (T1-F and T2-F) found on the forward strand and R3 is calculated using the highest two frequencies (T1-R and T2-R) found on the reverse strand. To calculate these two ratios, we divide the highest frequency by the second highest frequency T2. So R2 = (T1-F / T2-F) and R3 = (T1-R / T2-R). These two ratios are used to analyze termini characteristics on each strand taken individually. Li et al. suggested two possible interpretations for R2 and R3 ratios combine to R1. When R1 < 30 and R2 < 3, we either have no obvious termini on the forward strand, or we have multiple preferred termini on the forward strand, if 30 ≤ R1 ≤ 100. If R2 > 3, it is suggested that there is an obvious unique termini on the forward strand. The same reasoning is applicable for the result of R3. Combining the results for ratios found with this approach, it is possible to make the first prediction for the viral packaging mode of the analyzed phage. A unique obvious termini present at both ends (both R2 and R3 > 3) reveals the presence of a COS mode of packaging. The headful mode of packaging PAC is concluded when we have a single obvious termini only on one strand. A whole coverage around 500X is needed for this method to be reliable.

**Nearby Termini (Forward / Reverse)**	0 / 0	Peaks localized 20 bases around the maximum
**R1 - highest freq./average freq.**	281	At least one fixed termini is present with terminase recognizing a specific site.
**R2 Forw - highest freq./second freq.**	1	Multiple preferred termini on the forward strand.
**R3 Rev - highest freq./second freq.**	11	Unique termini on the reverse strand.
